# Olfactory function is associated with cognitive performance: results from the population-based LIFE-Adult-Study

**DOI:** 10.1186/s13195-019-0494-z

**Published:** 2019-05-10

**Authors:** Maryam Yahiaoui-Doktor, Tobias Luck, Steffi G. Riedel-Heller, Markus Loeffler, Kerstin Wirkner, Christoph Engel

**Affiliations:** 1 Institute for Medical Informatics, Statistics and Epidemiology, University of Leipzig, Haertelstrasse 16-18, 04109 Leipzig, Germany; 2 LIFE - Leipzig Research Centre for Civilization Diseases, University of Leipzig, Leipzig, Germany; 3 Department of Economic and Social Sciences and Institute of Social Medicine, Rehabilitation Sciences and Healthcare Research (ISRV), University of Applied Sciences Nordhausen, Nordhausen, Germany; 4 Institute of Social Medicine, Occupational Health and Public Health (ISAP), University of Leipzig, Leipzig, Germany

**Keywords:** Cognition, Olfactory function, General population, Cross-sectional

## Abstract

**Background:**

Studies in older adults or those with cognitive impairment have shown associations between cognitive and olfactory performance, but there are few population-based studies especially in younger adults. We therefore cross-sectionally analyzed this association using data from the population-based LIFE-Adult-Study.

**Methods:**

Cognitive assessments comprised tests from the Consortium to Establish a Registry for Alzheimer’s Disease (CERAD): verbal fluency (VF), word list learning and recall (WLL, WLR), and the Trail Making Tests (TMT) A and B. The “Sniffin’ Sticks Screening 12” test was used to measure olfactory performance. Linear regression analyses were performed to determine associations between the number of correctly identified odors (0 to 12) and the five cognitive test scores, adjusted for sex, age, education, and the presence of depressive symptoms. Receiver operating characteristic (ROC) analysis was carried out to determine the discriminative performance of the number of correctly identified odors regarding identification of cognition impairment.

**Results:**

A total of 6783 participants (51.3% female) completed the olfaction test and the VF test and TMT. A subgroup of 2227 participants (46.9% female) also completed the WLL and WLR tests. Based on age-, sex-, and education-specific norms from CERAD, the following numbers of participants were considered cognitively impaired: VF 759 (11.2%), WLL 242 (10.9%), WLR: 132 (5.9%), TMT-A 415 (6.1%), and TMT-B/A ratio 677 (10.0%). On average, score values for VF were higher by 0.42 points (*p* < 0.001), for WLL higher by 0.32 points (*p* = 0.001), for WLR higher by 0.31 points (*p* = 0.002), for TMT-A lower by 0.25 points (*p* < 0.001), and for TMT-B/A ratio lower by 0.01 points (*p* < 0.001) per number of correctly identified odors. ROC analysis revealed area under the curve values from 0.55 to 0.62 for the five cognitive tests.

**Conclusions:**

Better olfactory performance was associated with better cognitive performance in all five tests in adults — adjusted for age, sex, education, and the presence of depressive symptoms. However, the ability of the smell test to discriminate between individuals with and without cognitive impairment was limited. The value of olfactory testing in early screening for cognitive impairment should be investigated in longitudinal studies.

## Background

It has been suggested that both olfactory and certain cognitive functions are controlled via the orbitofrontal cortex and that poorer olfactory ability, as well as the manifestation of dementia, is associated with brain changes in the hippocampus and entorhinal cortex [1–5]. In further studies, pathological features of cognitive impairment have shown an association with olfactory dysfunction [2, 6, 7].

A direct association between cognitive impairment and olfactory performance has been shown previously, but in select groups: older adults, individuals already diagnosed with a cognitive impairment, or members of certain communities [8–18]. One study found that sensory impairment, which included olfaction, is associated with subtle deficits in cognitive function, a possible indicator of early brain aging [19]. Some have therefore suggested olfactory dysfunction could be a suitable biomarker for predicting cognitive impairment [8, 11–13, 20, 21]. A small number of population studies have investigated this topic, for instance Larsson et al. looked at middle-aged to older adults (45–90 years) and found an association between cognitive and olfactory performance [22]. Stanciu et al. focused on 1529 middle-aged to older adults from the same cohort and concluded olfactory assessment might supplement other assessments in evaluating the risk of conversion to dementia [23]. Roberts et al. focused their research on 1680 older adults (70–89 years) and concluded olfactory impairment is associated with cognitive impairment and testing it might have potential utility in screening [13]. Tebrügge et al. examined 2640 middle-aged to older adults (55–86 years) and concluded that the association between olfactory function and cognitive performance “may serve as a marker to improve identification of persons at high risk for cognitive decline and dementia” [24].

This body of evidence indicates a need to determine the value of olfactory testing as a possible predictor of cognitive impairment in unselected groups, i.e., not older or cognitively impaired individuals. This prompted us to investigate this topic using data from the LIFE-Adult-Study, a large population study of 10,000 randomly selected adults (aged 18–79) from a large city in Germany, using established cognitive and olfactory tests [25].

We used the “Sniffin’ Sticks” odor identification test for determining olfactory function, and five different cognitive tests: the Consortium to Establish a Registry for Alzheimer’s Disease (CERAD) word list learning (WLL), word list recall (WLR), and verbal fluency (VF) tests, and the Trail Making Test A (TMT-A) and Trail Making Test B, whereby the B/A ratio (TMT-B/A) was used in our analysis [26–28].

## Methods

### Study description

This study used data from the LIFE-Adult-Study, a population-based prospective cohort study of 10,000 participants conducted in Leipzig, Germany, by the Leipzig Research Centre for Civilization Diseases (First round of recruitment: August 2011 to November 2014). The aims of the LIFE-Adult-Study are to investigate the prevalence, early onset markers, genetic predispositions, and the role of lifestyle factors of major civilization diseases. The primary focus is on metabolic and vascular diseases, heart function, cognitive impairment, brain function, depression, sleep disorders and vigilance dysregulation, retinal and optical nerve degeneration, and allergies. The participants were randomly selected from the residents’ registration list and invited for voluntary study participation. The participation rate was 33%. The participants underwent a comprehensive assessment on the first day, while some were invited for further assessments on two additional days. The study was approved by the ethics committee of the University of Leipzig’s medical faculty, and complies with the ethical standards of the Declaration of Helsinki. Details of the study design have been published elsewhere [25].

### Testing of cognitive and olfactory performance

The cognitive tests chosen in this work cover several important cognitive domains, which can be affected in the early stages of a potential neurodegenerative process. TMT-A is used as a measure of attention or cognitive processing speed, while the TMT-B/A ratio (which was used in this analysis) is often used as measure for executive functioning. VF tests verbal abilities and semantic memory, while the WLL assesses the ability to learn new verbal information. The WLR test subsequently measures verbal memory and delayed free recall. For a more detailed description of these and other neurocognitive tests used in the LIFE-Adult-Study and their age-, sex-, and education-specific norms, see Luck et al. [29].

The “Sniffin’ Stick Screening 12” test (Burghart Messtechnik GmbH, Wedel, Germany) was used to determine olfactory function. This is a shorter version of the odor identification subset of the “Sniffin’ Stick” test battery, a commonly used and validated test set for olfactory function. The full test battery has three subsets of tests for determining odor discrimination, odor identification, and odor detection threshold [26, 27]. The subset used here consisted of 12 different common, everyday odors, which the participants had to identify one by one on a felt-pen. For each felt-pen, the participants were given a list of four choices of what the odor could be, of which they had to choose one (forced choice), and the total score ranges from 0 to 12 points (hereafter referred to as the “smell test score”). Normative data, the validity of the “Sniffin’ Stick Screening 12” test, its cultural adaptation, and suggestions for its use, including cut-off points for normosmia, have been published elsewhere [26, 27, 30–34]. For a more detailed description of the use of the “Sniffin’ Stick Screening 12” test in the LIFE-Adult-Study, see Hinz et al. [35].

The test was added to the study some time after the study has started; therefore, not all, but a total of 7381 participants from the 10,000, could take the test. Of these, 114 were excluded because the smell test was not completed. Of the remaining 7267 individuals, 73 were excluded because they had not completed all three tests: verbal fluency, Trail Making Test A, and Trail Making Test B/A. A further 395 participants were excluded due to missing information on education or depression. Of the remaining 6799, 16 were excluded due to a diagnosis of Parkinson’s disease, resulting in 6783 individuals available for the analysis. Of these 6783 participants, 2227 underwent further examinations consisting of deeper cognitive testing (tests Word list learning and Word list recall).

### Statistical analysis

Descriptive statistics were used to characterize the study population. The association between cognitive and smell test scores was first examined graphically where participants with smell test scores ≤ 8 points were put into one group. We looked at the discriminatory performance of the smell test for predicting cognitive impairment (defined as at least one standard deviation below age-, sex-, and education-specific norms from CERAD) for each of the cognitive tests using receiver operating characteristic (ROC) analysis. Sensitivities, specificities, and Youden’s indices were calculated for different cut-off values of the smell test score. The cognitive test scores were scalar; therefore, linear regression analysis was chosen to model the association between smell test scores (explanatory variable) and each of the five cognitive test scores (dependent variable). In the next step, the linear regression was adjusted for the presence of depressive symptoms (defined by a score of ≥ 23 in the Center for Epidemiologic Studies Depression Scale), age (continuous), university education (yes/no), and sex, as these attributes have been identified to affect cognitive function in current literature [36–43]. In order to make the regression coefficients comparable between the five models, cognitive score values were re-scaled to have a mean value of 50 and a standard deviation of 10.

Statistical analyses were carried out using R version 3.4.2 (R Core Team, 2017. R: A language and environment for statistical computing. R Foundation for Statistical Computing, Vienna, Austria. URL: https://www.r-project.org) and confirmed in SPSS Version 25 (IBM Corporation, New York, USA). The R function *glm* from the package *stats* with the method *glm.fit* was used for the linear regression analysis, as it is flexible, offering generalized linear modeling. For all analyses, an alpha level of 0.05 was used to determine statistical significance (two-tailed).

## Results

### Participant characteristics

A total of 6783 participants completed the smell test and the three cognitive tests VF, TMT-A, an TMT-B/A, of which 3483 (51%) were female (Table 1). Due to the special age focus of the LIFE-Adult-Study, the majority of participants were 40 years or older. Male participants were more often university-educated (33.2%) than female participants (24.9%). More females showed depressive symptoms (9.4% vs. 3.7%), while the median smell test score for males and females was the same (10, IQR, 9–11).

**Table 1:** Participant characteristics

	Female (*n* = 3483, 51.3%)	Male (*n* = 3300, 48.7%)	Total (*n* = 6783)
Age (years)			
18–29	97 (2.8%)	85 (2.6%)	182 (2.7%)
30–39	81 (2.3%)	111 (3.4%)	192 (2.8%)
40–49	993 (28.5%)	881 (26.7%)	1874 (27.6%)
50–59	870 (25.0%)	744 (22.5%)	1614 (23.8%)
60–69	812 (23.3%)	777 (23.5%)	1589 (23.0%)
70–79	630 (18.1%)	702 (21.3%)	1332 (19.6%)
Smell test score^*^	10 (9–11)	10 (9–11)	10 (9–11)
Depressive symptoms	327 (9.4%)	122 (3.7%)	449 (6.6%)
University education	869 (24.9%)	1097 (33.2%)	1966 (29.0%)
CERAD verbal fluency^*^	24.0 (20.0–28.0)	23.0 (19.0–28.0)	24.0 (19.0–28.0)
Higher values are better Cognitively impaired^#^	398 (11.4%)	361 (10.9%)	759 (11.2%)
Trail Making Test A^*^	33.0 (26.0–42.0)	34.0 (27.0–44.0)	34.0 (26.0–43.0)
Max score 180			
Lower values are better			
Cognitively impaired^#^	208 (6.0%)	207 (6.3%)	415 (6.1%)
Trail Making Test B/A^*^	2.2 (1.8–2.8)	2.3 (1.9–2.8)	2.3 (1.9–2.8)
Lower values are better			
Cognitively impaired^#^	276 (7.9%)	401 (12.2%)	677 (10.0%)

*Median (IQR)

#At least one standard deviation below age-, sex-, and education-specific norms from CERAD

In the subgroup who also completed the two tests WLL and WLR, 1044 individuals (46.9%) were female (Table 2). The majority of participants in this subgroup were 60 years or older. Male participants were university-educated (39.7%) more often than female participants (24.4%), while the median smell test score for males and females was the same (10, IQR, 9–11).

**Table 2:** Participant characteristics (subgroup)

	Female (*n* = 1044, 46.9%)	Male (*n* = 1183, 53.1%)	Total (*n* = 2227)
Age (years)			
18–29	83 (8.0%)	72 (6.1%)	155 (7.0%)
30–39	58 (5.6%)	89 (7.5%)	147 (6.6%)
40–49	67 (6.4%)	88 (7.4%)	155 (7.0%)
50–59	28 (2.7%)	27 (2.3%)	55 (2.5%)
60–69	411 (39.4%)	430 (36.3%)	841 (37.8%)
70–79	397 (38.0%)	477 (40.3%)	874 (39.2%)
Smell test score^*^	10 (9–11)	10 (9–11)	10 (9–11)
Depressive symptoms	83 (8.0%)	40 (3.4%)	123 (5.5%)
University education	255 (24.4%)	470 (39.7%)	725 (32.6%)
CERAD word list learning^*^	24.0 (21.0–26.0)	22.0 (19.0–25.0)	23.0 (20.0–25.0)
Max score 30			
Higher values are better Cognitively impaired^#^	136 (13.0%)	106 (9.0%)	242 (10.9%)
CERAD word list recall^*^	9.0 (7.0–10.0)	8.0 (7.0–9.0)	8.0 (7.0–9.0)
Max score 10			
Higher values are better			
Cognitively impaired^#^	76 (7.3%)	56 (4.7%)	132 (5.9%)

*Median (IQR)

#At least one standard deviation below age-, sex-, and education-specific norms from CERAD

Females had better median scores in four of the five cognitive tests. The proportion of participants that would be categorized as cognitively impaired using CERAD norms was similar for females and males except for the TMT-B/A (7.9% v 12.2% respectively) and WLR (7.3% v 4.7%).

### Smell test scores and cognitive performance

**Fig. 1:**
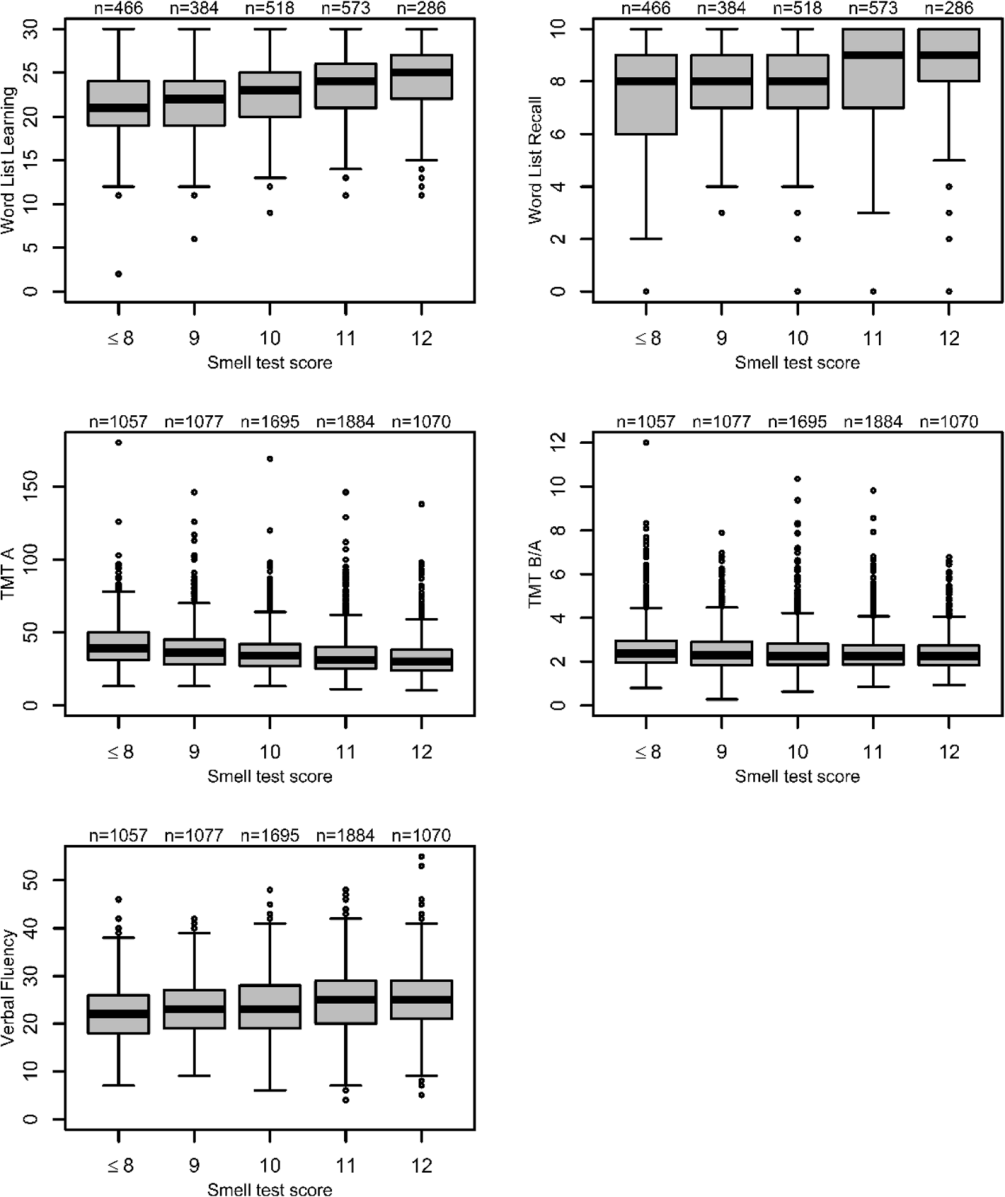
Association of olfactory performance and cognitive performance

For all cognitive tests, the score values were examined dependent on the olfactory performance, as shown in Fig. 1. The cognitive test scores were better with higher values of the smell test score, which was observed across all tests.

Table 3 shows the results of the univariable and multivariable linear regression analyses. Better smell test scores were significantly associated with better cognitive test results for all analyses (univariable and multivariable). Age, education, and sex were also significantly associated with cognitive test scores across the board. Depression was significantly associated with three of the tests (VF, TMT-A, and TMT-B/A). In the multivariable analyses (adjusted for sex, age, university education, depressive symptoms), the strongest and weakest associations for the smell test score were with VF and TMT-B/A (*β* 0.42 and − 0.01 respectively) and in the univariable analyses with TMT-A and TMT-B/A (*β* − 1.24 and − 0.16 respectively).

**Table 3:** Association of smell test scores with cognitive performance

CERAD test		Univariable analysis		Multivariable analysis	
	Factor	Regression coefficient (95%CI)	*p* value	Regression coefficient (95%CI)	*p* value
Verbal fluency *n* = 6783	Smell test score	0.79 (0.65–0.92)	*< 0.001*	0.42 (0.28–0.56)	*< 0.001*
	Depressive symptoms	− 1.27 (−2.22 to −0.32)	*0.009*	− 1.42 (−2.35 to − 0.49)	*0.003*
	Age	− 0.14 (− 0.16 to − 0.12)	*< 0.001*	− 0.12 (− 0.14 to − 0.10)	*< 0.001*
	University education	4.05 (3.54–4.57)	*< 0.001*	4.03 (3.54–4.55)	*< 0.001*
	Female sex	1.01 (0.54–1.49)	*< 0.001*	1.19 (0.72–1.65)	*< 0.001*
Word list learning *n* = 2227	Smell test score	1.19 (0.99–1.39)	*< 0.001*	0.32 (0.13–0.50)	*0.001*
	Depressive symptoms	0.64 (− 1.06–2.35)	0.459	− 0.86 (− 2.30–0.59)	0.245
	Age	− 0.30 (− 0.32 to − 0.28)	*< 0.001*	− 0.28 (− 0.31 to − 0.26)	*< 0.001*
	University education	2.70 (1.88–3.52)	*< 0.001*	2.84 (2.13–3.55)	*< 0.001*
	Female sex	3.57 (2.80–4.33)	*< 0.001*	3.84 (3.17–4.51)	*< 0.001*
Word list recall *n* = 2227	Smell test score	1.06 (0.86–1.27)	*< 0.001*	0.31 (0.12–0.51)	*0.002*
	Depressive symptoms	1.70 (− 0.02–3.43)	0.053	0.38 (− 1.16–1.92)	0.624
	Age	− 0.26 (− 0.29 to − 0.24)	*< 0.001*	− 0.25 (− 0.27 to − 0.22)	*< 0.001*
	University education	2.18 (1.34–3.01)	*< 0.001*	2.32 (1.56–3.08)	*< 0.001*
	Female sex	3.23 (2.45–4.01)	*< 0.001*	3.38 (2.66–4.09)	*< 0.001*
Trail Making Test A *n* = 6783	Smell test score	− 1.12 (− 1.24 to − 1.00)	*< 0.001*	− 0.25 (− 0.36 to − 0.13)	*< 0.001*
	Depressive symptoms	1.41 (0.53–2.28)	*0.002*	2.23 (1.47–2.99)	*< 0.001*
	Age	0.37 (0.35–0.38)	*< 0.001*	0.36 (0.34–0.37)	*< 0.001*
	University education	− 0.84 (− 1.32 to − 0.36)	*< 0.001*	−0.78 (− 1.19 to − 0.36)	*< 0.001*
	Female sex	−1.19 (− 1.62 to − 0.76)	*< 0.001*	−1.01 (− 1.39 to − 0.63)	*< 0.001*
Trail Making Test B/A *n* = 6783	Smell test score	− 0.16 (− 0.21 to − 0.11)	*< 0.001*	−0.01 (− 0.15 to − 0.04)	*< 0.001*
	Depressive symptoms	0.42 (0.06–0.77)	*0.022*	0.44 (0.09–0.80)	*0.015*
	Age	0.02 (0.02–0.03)	*< 0.001*	0.02 (0.01–0.03)	*< 0.001*
	University education	− 1.07 (− 1.27 to − 0.88)	*< 0.001*	− 1.08 (− 1.28 to − 0.89)	*< 0.001*
	Female sex	− 0.30 (− 0.47 to − 0.12)	*0.001*	− 0.36 (− 0.54 to − 0.12)	*< 0.001*

values in italics are statistically significant

### Analysis of the discriminatory performance of the smell test

In Table 4, the results of the ROC analysis are shown. The value of the area under the ROC curve (AUC), which is a measure of how well the olfaction score is able to discriminate between cognitively impaired and cognitively healthy individuals, was highest for TMT-A (0.62) and lowest for VF and TMT-B/A (0.55). Youden’s indices, which can be used to determine an optimal cut-off value of the smell test score for the best discrimination using their highest value (i.e., the maximum of the sum of sensitivity and specificity), suggest that an optimal cut-off value is < 10 for WLL, WLR, and TMT-A and < 11 for VF and TMT-B/A.

**Table 4:** Results of ROC analysis

Cognitive test		Smell test score cut-off*					
		< 8	< 9	< 10	< 11	< 12	AUC (95% CI)
VF	Sensitivity	10.4	19.0	37.8	63.0	86.4	0.55
	Specificity	92.1	84.8	69.3	44.4	16.1	(0.52–0.57)
	Youden’s index	2.5	3.8	7.1	*7.4*	2.5	*p <* 0.001
WLL	Sensitivity	15.7	28.1	50.0	71.5	92.6	0.58
	Specificity	89.4	79.9	63.3	39.8	13.5	(0.55–0.62)
	Youden’s index	5.1	8.0	*13.3*	11.3	6.1	*p <* 0.001
WLR	Sensitivity	15.9	32.6	49.2	72.0	93.2	0.59
	Specificity	89.1	79.8	62.5	39.2	13.2	(0.54–0.64)
	Youden’s index	5.0	12.4	*11.7*	9.4	6.4	*p =* 0.001
TMT-A	Sensitivity	14.9	29.6	50.4	71.3	90.4	0.62
	Specificity	92.2	85.3	69.8	44.5	16.2	(0.59–0.65)
	Youden’s index	7.1	14.9	*20.2*	15.8	6.6	*p <* 0.001
TMT-B/A	Sensitivity	10.5	19.1	38.3	63.8	88.2	0.55
	Specificity	92.1	84.8	69.3	44.4	16.2	(0.53–0.58)
	Youden’s index	2.6	3.9	7.6	*8.2*	4.4	*p <* 0.001

Youden’s index = sensitivity + specificity − 1. Highest Youden’s index is indicated in italics

Sensitivity, specificity, and Youden’s index are given as percentages

*Individuals having a smell test score below the cut-off are “test positive”, i.e., suspected of being cognitively impaired

## Discussion

In this study, we investigated the association between olfactory and cognitive performance, to the best of our knowledge, in the largest population-based sample to date with a broad age range that does not have a specifically higher risk of cognitive impairment. We could show that higher smell test scores were significantly associated with better verbal abilities and semantic memory (VF), better ability to learn new verbal information (WLL), verbal memory and delayed free recall (WLR), attention or cognitive processing speed (TMT-A), and executive function (TMT-B/A), after adjustment for age, sex, education, and depressive symptoms.

Our results support the findings of other studies that the association of olfactory and cognitive performance goes beyond the effects of aging [1, 8, 12, 14, 20]. Other population studies that investigated this topic, albeit in older or at risk of developing cognitive impairment populations, came to the same conclusion [13, 22–24]. Our analysis extends these analyses by additionally controlling for depressive symptoms, which may be a risk factor for later life dementia [37].

Larrson et al. found a significant association between cognitive speed and vocabulary, and odor identification performance, having controlled for age, sex, and education [22]. We also found olfactory performance to be significantly associated with both cognitive speed and vocabulary. Their tasks measuring executive functioning (tower of Hanoi) found no association with odor identification performance, whereas we found a statistically significant association between olfactory performance and executive function (TMT-B/A). This difference could be due to the different methods used to determine executive function. A later analysis of the data from the above study found that olfactory impairment was a predictor of dementia in older adults (sample mean age 61 ± 11.7, dementia group mean age 75.5 ± 7.4) [23].

In the population-based Mayo Clinic study of Aging, Roberts et al. found a significant association between worsening olfactory and cognitive performance having adjusted for sex and education in older adults (mean age 79.5 ± 5.3) [13]. This matched our findings, albeit their population was older. Tebrügge et al. evaluated sex- and age-specific associations of olfactory and cognitive performance in a population-based study of 2640 participants aged 55–86 years [24]. They found anosmics performed worst in their cognitive tests and normosmics best, for both sexes, in the age group 65–74. Two of their eight cognitive tests were the same as ours, VF and TMT-A, and our results agree with theirs. Again, our results matched their findings, while their population tended to be older.

A growing number of biophysical investigations into this topic support the use of olfactory testing as a screening method for cognitive decline. Marigliano et al. for instance suggested in 2014 olfactory testing, in comparison to measuring hippocampal volume loss, has potential utility in early detection of Alzheimer’s disease [1]. In 2015, Growdon et al. concluded that olfactory testing has the potential to contribute to detecting pre-clinical Alzheimer’s disease in clinically normal individuals, having associated it with entorhinal cortex thickness [2].

However, the clinical use of these findings is not yet fully agreed. For instance, Eibenstein et al. suggested in 2005 that olfactory testing should be part of the diagnostic armamentarium of pre-clinical dementia [8]. Later, in 2009, Laasko et al. emphasized that standard assessment of olfactory nerve function is not sufficient to study cognitive impairment in MCI [9]. More recently, however, support has gathered for using olfactory assessment as a tool for early detection of cognitive impairment. For instance, Sohrabi et al. concluded in their 2012 study that their results indicate that impairment in olfactory discrimination can predict future cognitive decline, a case for early screening use of olfactory testing [16]. Devanand et al. suggested the use of odor identification tests as an early marker of cognitive decline, in the clinical setting, or for further treatment research in their 2015 study [20]. Streit et al. concluded a smell test could be of benefit in cases of suspected dementia, but together with neurocognitive tests in their 2015 study [14].

While olfaction tests do seem to be easy and relatively cheap to perform, olfactory identification tests alone may be insufficient or not sensitive enough as a predictor of cognitive impairment [9, 10]. A combination of the common cognitive diagnostic tools and an olfaction test has been put forward as a better way to detect cognitive impairment, preferably at earlier stages [14, 15]. Several studies from 2016 also concluded olfactory testing would be useful in the detection of cognitive decline. Ottaviano et al. recommended that “elderly patients complaining of smell loss and found to be dysosmic, by means of validated olfactory tests, should be neurologically evaluated as early as possible to detect slight motor abnormalities in an at-risk population” [11]. Roalf et al. concluded that “a simple-to-administer test of odor identification warrants inclusion in the screening of individuals at risk for developing Alzheimer’s disease” [12]. Roberts et al. suggested that olfactory tests have potential utility for screening for MCI and MCI that is likely to progress [13]. Wongrakpanich et al. recommended early detection of sensorineural dysfunction by history, physical examination, and screening tests [15]. In our study, we investigated whether optimal cut-off points for the smell test score could be determined in order to identify individuals with cognitive impairment. Using ROC analysis, we found optimal cut-off points—which maximize both specificity and sensitivity (as expressed by Youden’s index)—in the region of 10 and 11 score points. This matches the cut-off points found in the literature that distinguish between normosmia and dysosmia, albeit that the discriminatory power that we saw in our analysis is generally low. It must however here be noted that the full range of the “Sniffin’ Sticks” test battery was not administered, which means testing of olfactory thresholds and suprathreshold odor discrimination was not carried out. It remains to be shown whether this would improve the discriminatory performance.

Our study has some limitations. Firstly, our data did not include a history of head trauma, history of sinusitis nor active respiratory illness, which could have had an impact on olfactory function and thus mask the association between olfactory and cognitive performance. Secondly, the proportion of participants ≥ 60 was higher in the subgroup with the tests WLL and WLR (*n* = 2227), which may make the analysis results less comparable to the main group (*n* = 6783). Lastly, the smell test we used is a naming test, which means that naming deficits due to cognitive impairment but independent of olfactory performance may influence the score. Since naming deficits were not measured in our study, we could not determine the extent to which this could have impacted the results.

## Conclusions

We found a consistently significant association of the olfactory performance with cognitive performance in our cross-sectional study. However, the performance of the odor identification test alone to discriminate between individuals with and without cognitive impairment was limited. Further studies are needed to evaluate whether deeper testing of olfactory performance, e.g., using the full Sniffin’ Sticks test battery improves discrimination. Moreover, longitudinal studies are needed to show whether and which methods of olfactory testing are suitable to predict the risk of cognitive impairment.

## Abbreviations

CERAD: Consortium to Establish a Registry for Alzheimer’s Disease
ERDF: European Regional Development Fund
TMT: Trail Making Tests
VF: Verbal fluency
WLL: Word list learning
WLR: Word list recall
